# Mycobacterium tuberculosis Beijing genotype emerging in Vietnam.

**DOI:** 10.3201/eid0603.000312

**Published:** 2000

**Authors:** D D Anh, M W Borgdorff, L N Van, N T Lan, T van Gorkom, K Kremer, D van Soolingen

**Affiliations:** National Institute of Hygiene and Epidemiology, Hanoi, Vietnam.

## Abstract

To assess whether the Mycobacterium tuberculosis Beijing genotype is emerging in Vietnam, we analyzed 563 isolates from new cases by spoligotyping and examined the association between the genotype and age, resistance, and BCG vaccination status. Three hundred one (54%) patients were infected with Beijing genotype strains. The genotype was associated with younger age (and hence with active transmission) and with isoniazid and streptomycin resistance, but not with BCG vaccination.


### *Mycobacterium tuberculosis* Beijing Genotype Emerging in Vietnam

**Dang Duc Anh,* Martien W. Borgdorff,† L.N. Van,‡ N.T.N. Lan,§ Tamara van Gorkom,¶ Kristin Kremer,¶ and Dick van Soolingen¶**
*National Institute of Hygiene and Epidemiology, Hanoi, Vietnam; †Royal Netherlands Tuberculosis Association, The Hague, The Netherlands; ‡National Institute of Tuberculosis and Respiratory Diseases, Hanoi, Vietnam; §Tuberculosis and Lung Diseases Center, Ho Chi Minh City, Vietnam; and ¶National Institute of Public Health and the Environment, Bilthoven, The Netherlands

To assess whether the ***Mycobacterium tuberculosis*** Beijing genotype is emerging in Vietnam, we analyzed 563 isolates from new cases by spoligotyping and examined the association between the genotype and age, resistance, and BCG vaccination status. Three hundred one (54%) patients were infected with Beijing genotype strains. The genotype was associated with younger age (and hence with active transmission) and with isoniazid and streptomycin resistance, but not with BCG vaccination.

A high degree of diversity of ***Mycobacterium tuberculosis*** has been shown with restriction fragment length polymorphism (RFLP) typing using IS***6110*** as a probe, particularly in countries like the Netherlands, where many tuberculosis (TB) cases occur among immigrants (1). However, in the Beijing region of China, a particular genotype was found in >80% of the TB patients and was thus designated the Beijing genotype (2). In other parts of China and in Asian countries such as Mongolia, Thailand, and Korea, 40% to 50% of the tested ***M. tuberculosis*** isolates represented this genotype (2). Although Beijing genotype strains carry a large number of IS***6110*** insertion elements, the IS***6110*** RFLP patterns are highly similar (2). Moreover, the spoligopatterns of Beijing genotype strains are identical and distinct from those of other ***M. tuberculosis*** strains (1), which suggests that strains of the Beijing genotype have emerged recently from a single ancestor.

Reasons for the predominance of a narrow range of genotypes may include limited contact with other populations or a selective advantage of certain strains due to reduced sensitivity to vaccine-induced immunity. For instance, wide-scale application of vaccines against whooping cough (***Bordetella pertussis***) has led to shifts in the populations of circulating pathogens (3). BCG vaccination, which has been applied widely in China since the early 1950s, may have led to a similar shift in the population of ***M. tuberculosis***. Alternatively, a selective advantage may be provided by reduced sensitivity to anti-TB drugs. The Beijing genotype was associated with recent transmission of drug-resistant strains in Cuba, Germany, Russia, and Estonia (4-7). The largest known epidemic of multidrug-resistant TB in North America was caused by the “W” strain, a variant of the Beijing genotype (8).

Vietnam is one of 22 countries in which 80% of the world’s new TB cases occur (9). Among these 22 countries, Vietnam has one of the most successful directly observed therapy short-course programs, with a cure rate of approximately 90% and a case-detection rate estimated at 67% in 1996 and at >70% since then (National Tuberculosis Programme, unpub. data). BCG coverage has been high (>80%) during the past decade, and the level of primary multidrug resistance was recently estimated at 2.3% (9,10). We investigated the spread of ***M. tuberculosis*** Beijing genotype strains in Vietnam and whether the spread is associated with BCG vaccination status or drug resistance.

Address for correspondence: D. van Soolingen, Diagnostic Laboratory for Infectious Diseases and Perinatal Screening, National Institute of Public Health and the Environment, P.O. Box 1, 3720 BA Bilthoven, The Netherlands; fax: 31-30-2744418; e-mail: d.van.soolingen@rivm.nl.

In total, 822 isolates were obtained from TB patients whose age and BCG status were known. Of these, 563 had newly diagnosed disease (Table 1). The isolates were collected in 1998 and the first quarter of 1999 at the Tuberculosis and Lung Diseases Centre in Ho Chi Minh City and the National Institute of Tuberculosis and Respiratory Diseases in Hanoi, respectively. The isolates were analyzed by spoligotyping (11). In spoligotyping, the genomic direct repeat (DR) region of ***M. tuberculosis*** complex bacteria is amplified by polymerase chain reaction (PCR) and the presence of 43 spacer sequences between the DRs is examined in a reversed line-blot assay (2,11). In previous studies, this method has proven highly reliable for distinguishing Beijing genotype strains (2). Among the 563 spoligopatterns analyzed, two predominant genotypes were recognized (Figure), of which the Beijing type was the most frequent (n = 301; 53%). The second most frequent genotype was not found in the database of spoligopatterns of 2,500 ***M. tuberculosis*** isolates from countries all over the world, held at the National Institute of Public Health and the Environment in Bilthoven and designated Vietnam genotype (n = 152; 27%) (Figure). The remaining 110 isolates exhibited 18 different spoligopatterns.

The Beijing genotype was strongly associated with younger age ($X^2_{trend}$; p <0.001), but not with BCG status, after the data were adjusted for age (Table 1). As levels of drug resistance varied between Hanoi and Ho Chi Minh City and the number of samples from Hanoi was small, the association of the Beijing genotype and drug resistance was restricted to Ho Chi Minh City (Table 2). Drug resistance was found more commonly in the Beijing than in other genotypes (Table 2). The association with drug resistance was significant for isoniazid (OR 1.7; 95% CI 1.1-2.6) and streptomycin (odds ratio [OR] 3.1; 95% confidence interval [CI] 2.0-4.6) resistance.

TB occurs partly as primary disease (typically defined as occurring within 5 years of infection) and partly as endogenous reactivation or exogenous reinfection (occurring >5 years

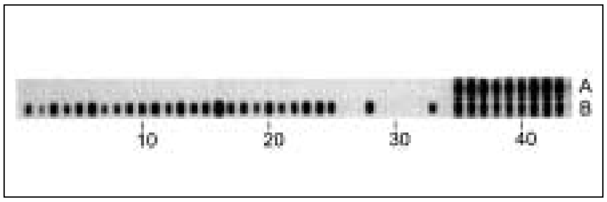

Figure. Representative spoligotype patterns of the Beijing (A) and the Vietnam (B) genotypes. Numbers indicate the spacer oligonucleotide sequences, present on the reversed line blot, which are derived from reference ***Mycobacterium tuberculosis*** strain H37Rv and ***M. bovis*** BCG vaccine strain P3.

Table 1. Beijing genotype of ***Mycobacterium tuberculosis*** in 563 new tuberculosis cases, by age and BCG status, in Hanoi and Ho Chi Minh City, Vietnam, 1998

| | | Beijing genotype | | OR[a] (95% CI[b]) | |
|---|---|---|---|---|---|
| | No. | No. | (%) | Crude | Adjusted[c] |
| Province | | | | | |
| Hanoi | 64 | 37 | (58) | 1 (p>0.05) | |
| Ho Chi Minh City | 499 | 264 | (53) | 0.8 (0.5-1.4) | |
| Age group | | | | | |
| <25 | 76 | 54 | (71) | 1 (p<0.001) | 1 |
| 25-34 | 173 | 102 | (59) | 0.6 (0.3-1.0) | 0.6 (0.3-1.1) |
| 35-44 | 164 | 83 | (51) | 0.4 (0.2-0.7) | 0.4 (0.2-0.8) |
| 45-54 | 74 | 31 | (42) | 0.3 (0.1-0.6) | 0.3 (0.2-0.6) |
| 55-64 | 39 | 16 | (41) | 0.3 (0.1-0.6) | 0.3 (0.1-0.7) |
| 65+ | 37 | 15 | (41) | 0.3 (0.1-0.6) | 0.3 (0.1-0.7) |
| BCG scar | | | | | |
| Yes | 285 | 167 | (59) | 1(p<0.05) | 1 |
| No | 278 | 134 | (48) | 0.7 (0.5-0.9) | 0.9 (0.6-1.3) |
| Total | 563 | 301 | (54) | | |

[a]OR, odds ratio.
[b]CI, confidence interval.
[c]Adjusted for age and BCG vaccination.

Table 2. Risk factors for drug resistance in 499 new tuberculosis cases in Ho Chi Minh City

| | | Resistance (%) | | | | | OR[a] (95% CI[b]) | |
|---|---|---|---|---|---|---|---|---|
| | No. | INH | SM | RIF | EMB | MDR | INH | SM |
| Genotype | | | | | | | | |
| Beijing | 264 | 28 | 42 | 3 | 3 | 3 | 1.7 (1.1-2.6) | 3.1 (2.0-4.6) |
| Other | 235 | 19 | 19 | 2 | 1 | 2 | 1 ($p<0.05$) | 1 ($p<0.001$) |
| Age group | | | | | | | | |
| <25 | 66 | 17 | 36 | 3 | 0 | 3 | 1 ($p>0.05$) | 1 ($p>0.05$) |
| 25-34 | 165 | 27 | 32 | 3 | 2 | 3 | 1.8 (0.9-3.8) | 0.8 (0.4-1.5) |
| 35-44 | 147 | 25 | 33 | 1 | 1 | 1 | 1.7 (0.8-3.5) | 0.8 (0.5-1.6) |
| 45-54 | 59 | 19 | 25 | 3 | 5 | 3 | 1.1 (0.5-2.9) | 0.6 (0.3-1.3) |
| 55-64 | 30 | 23 | 23 | 3 | 3 | 3 | 1.5 (0.5-4.4) | 0.5 (0.2-1.4) |
| 65+ | 32 | 31 | 31 | 0 | 0 | 0 | 2.3 (0.8-6.1) | 0.8 (0.3-2.0) |
| BCG scar | | | | | | | | |
| Yes | 259 | 22 | 31 | 3 | 2 | 3 | 1 ($p>0.05$) | 1 ($p>0.05$) |
| No | 402 | 73 | 22 | 2 | 2 | 2 | 1.3 (0.9-2.0) | 1.1 (0.7-1.6) |
| Total | 499 | 24 | 31 | 2 | 2 | 2 | | |

[a]OR, odds ratio.
[b]CI, confidence interval.
INH, isoniazide; SM, streptomycin; RIF, rifampin; EMB, ethambutol; MDR, multidrug resistant after infection). With increasing age, a decreasing proportion of cases is due to primary TB. Thus, the association of the Beijing genotype and young age suggests a recent spread of the Beijing genotype in Vietnam. The study of Qian et al., in which spoligotyping was performed on paraffin-embedded material, indicated that the Beijing genotype was presumably already prevalent in the Beijing region 30 to 40 years ago (12).

In Vietnam, the Beijing genotype occurs more commonly in those with a BCG scar than in those without it. However, this is likely to represent a cohort effect of BCG vaccination, rather than reduced sensitivity to vaccine-induced immunity of Beijing genotype strains. Because of increasing BCG vaccination coverage in Vietnam over the past 2 decades, young people are more likely to be vaccinated than older people. Within age groups, occurrence of the Beijing genotype is not associated with BCG vaccination status.

Its striking association with anti-TB drug resistance may explain the Beijing genotype's predominance in recently infected patients. Anti-TB drugs are widely used in Vietnam, and anti-TB drug resistance would thus provide a selective advantage. In vitro experiments should determine whether Beijing genotype strains have an increased intrinsic resistance to anti-TB drugs or an enhanced capacity to gain resistance against these drugs.

Dr. Anh is head of the Department of Bacteriology, National Institute of Hygiene and Epidemiology, Hanoi, Vietnam. Areas of interest include molecular epidemiology of mycobacterial infection and epidemiologic surveillance of bacterial meningitis in children.

### References

1. Van Soolingen D, Borgdorff MW, de Haas PEW, Kremer K, Veen J, Sebek MGG, et al. Molecular epidemiology of tuberculosis in The Netherlands: a nationwide study during 1993 through 1997. J Infect Dis 1999;180:726-36.
2. Van Soolingen D, Qian L, de Haas PEW, Douglas JT, Traore H, Portaels F. Predominance of a single genotype of ***Mycobacterium tuberculosis*** in countries of East Asia. J Clin Microbiol 1995;33:3234-8.
3. Van Loo IHM, van der Heide HGJ, Nagelkerke NJD, Mooi FR. Temporal trends in the population structure of ***Bordetella pertussis*** during 1949-1996 in a highly vaccinated population. J Infect Dis 1999;179:915-23.
4. Diaz R, Kremer K, de Haas PEW, Gomez RI, Marrero A, Valdivia JA. Molecular epidemiology of tuberculosis in Cuba outside of Havana, July 1994-June 1995: utility of spoligotyping versus IS***6110*** restriction fragment length polymorphism. Int J Tuberc Lung Dis 1998;2:743-50.
5. Niemann S, Rüsch-Gergdes S, Richter E. IS***6110*** fingerprinting of drug-resistant ***Mycobacterium tuberculosis*** strains isolated in Germany during 1995. J Clin Microbiol 1997;35:3016-20.
6. Martilla HJ, Soini H, Eerola E, Vyshnevskaya E, Vyshnevskiy BI, Otten TF. A Ser315Thr substitution in ***katG*** is predominant in genetically heterogeneous multidrug-resistant ***Mycobacterium tuberculosis*** isolates originating from the St. Petersburg area in Russia. Antimicrobiol Agents Chemother 1998;42:2443-5.

7. Ghebremichael S, Kruuner A, Levina K, Kallenius G, Hoffner SE. Molecular epidemiology of multi-drug resistant (MDR) tuberculosis in Estonia. Abstracts of the 19th Annual Congress of the European Society of Mycobacteriology. 1998; IV: 334.
8. Bifani PJ, Plikaytis BB, Kapur V, Stockbauer K, Pan X, Lutfey ML. Origin and interstate spread of a New York City multidrug-resistant ***Mycobacterium tuberculosis*** clone family. JAMA 1996;275:452-7.
9. Netto EM, Dye C, Raviglione MC. Progress in global tuberculosis control 1995-1996, with emphasis on 22 high-incidence countries. Int J Tuberc Lung Dis 1999;3:310-20.
10. World Health Organization/International Union Against Tuberculosis and Lung Disease global project on anti-tuberculosis drug resistance surveillance, 1994-1997. Anti-tuberculosis drug resistance in the world. Geneva: The Organization; 1997.
11. Kamerbeek J, Schouls LM, Kolk A, Van Agterveld M, van Soolingen D, Kuijper S. Simultaneous detection and strain differentiation of ***Mycobacterium tuberculosis*** for diagnosis and epidemiology. J Clin Microbiol 1997;35:907-14.
12. Qian LS, Van-Embden JDA, Van der Zanden AGM, Weltevreden EF, Duanmu H, Douglas JT. Retrospective analysis of the Beijing family of ***Mycobacterium tuberculosis*** in preserved lung tissues. J Clin Microbiol 1999;37:471-4.
